# The Future of the Oceans Past: Towards a Global Marine Historical Research Initiative

**DOI:** 10.1371/journal.pone.0101466

**Published:** 2014-07-02

**Authors:** Kathleen Schwerdtner Máñez, Poul Holm, Louise Blight, Marta Coll, Alison MacDiarmid, Henn Ojaveer, Bo Poulsen, Malcolm Tull

**Affiliations:** 1 Department of Social Sciences, Leibniz Center for Tropical Marine Ecology, Bremen, Germany; 2 Asia Research Center, Murdoch University, Murdoch, Western Australia, Australia; 3 Trinity Long Room Hub Arts and Humanities Research Institute, Trinity College Dublin, Dublin, Ireland; 4 World Wildlife Fund (WWF) -Canada, Vancouver, British Columbia, Canada; 5 Renewable Marine Resources Department, Institute of Marine Science, Barcelona, Spain; 6 Laboratoire Écosystèmes Marins Exploités, Sète Cedex, France; 7 Marine Ecology, National Institute of Water and Atmospheric Research, Kilbirnie, Wellington, New Zealand; 8 Estonian Marine Institute, University Tartu, Tartu, Estonia; 9 Faculty of Social Sciences, Aalborg University, Aalborg Ø, Denmark; 10 Murdoch Business School, Murdoch University, Murdoch, Western Australia, Australia; Seagrass Ecosystem Research Group, Swansea University, United Kingdom

## Abstract

Historical research is playing an increasingly important role in marine sciences. Historical data are also used in policy making and marine resource management, and have helped to address the issue of shifting baselines for numerous species and ecosystems. Although many important research questions still remain unanswered, tremendous developments in conceptual and methodological approaches are expected to contribute to a comprehensive understanding of the global history of human interactions with life in the seas. Based on our experiences and knowledge from the “History of Marine Animal Populations” project, this paper identifies the emerging research topics for future historical marine research. It elaborates on concepts and tools which are expected to play a major role in answering these questions, and identifies geographical regions which deserve future attention from marine environmental historians and historical ecologists.

## Introduction

In the last fifteen years marine science has taken a historical turn. Looking much further back in time than most previous studies, global initiatives such as the History of Marine Animal Populations (HMAP) and the Sea Around Us programmes, as well as individual studies have investigated diverse socio-ecological systems, from coastal European marshes to Pacific islands. In a parallel move, environmental historians have undertaken the study of human engagement with the underwater realm in a sea change for history. Historians, archaeologists, economists, sociologists and geographers, have engaged with marine scientists in an interdisciplinary effort to bring together the study of human and underwater worlds. Thanks to this collaborative effort of marine and human sciences, researchers have not only identified but, for many regions and species, resolved the problem of shifting baselines (a term popularised by Daniel Pauly in his seminal 1995 paper [1]) by pushing back the chronological limits of our knowledge [2].

**Figure 1:**
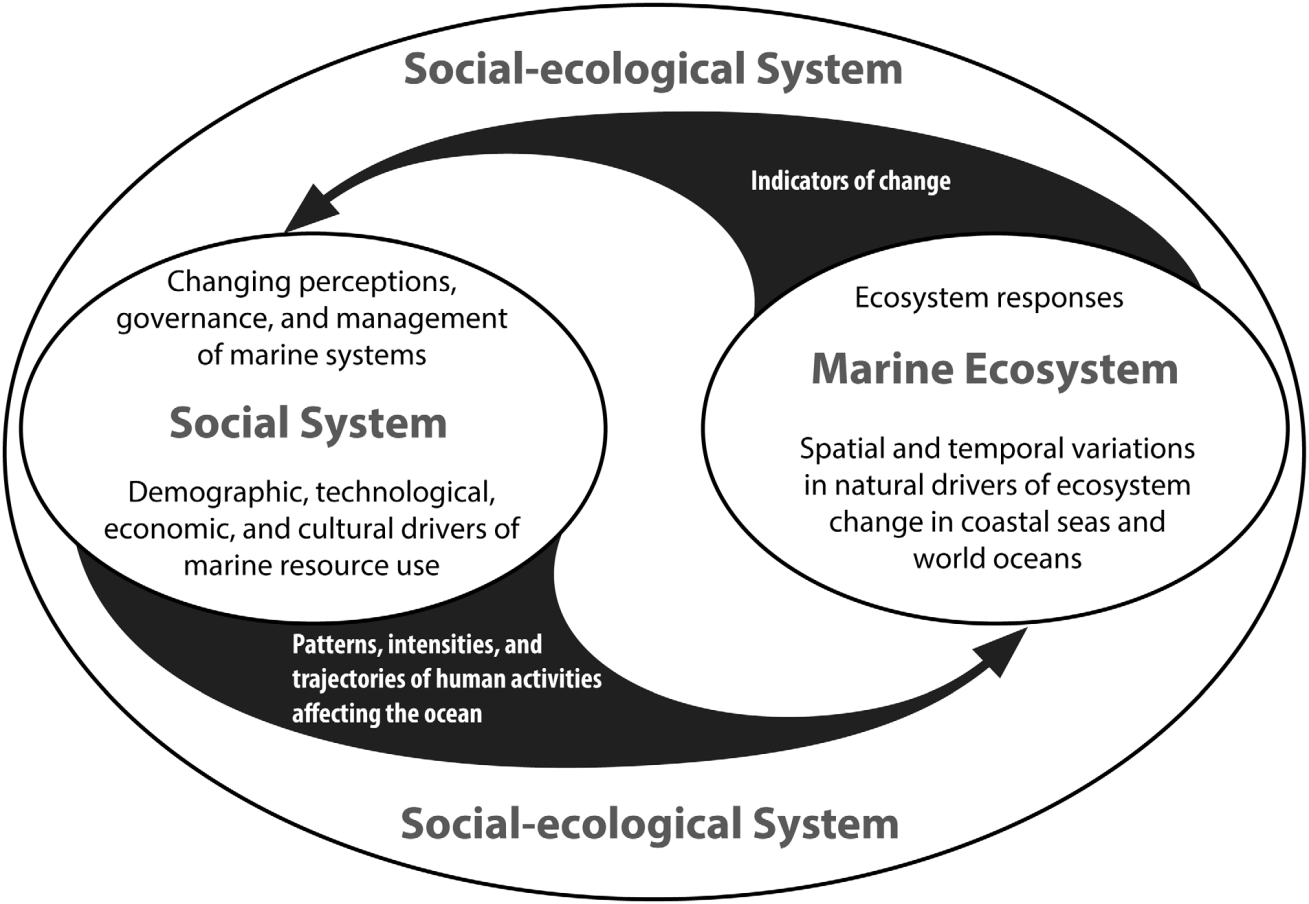
Integrated view of the global history of human interactions with life in the oceans.

While there have been tremendous advancements in marine historical research, these are yet to be assimilated into an integrated seamless view of the global history of human interactions with life in the oceans (Figure 1). Furthermore, many important questions still remain unanswered. The following are what we consider to be the five key areas of research inquiry for a global marine historical research agenda:

- What did the sea look like before human exploitations? So far, almost all the accessible information relates to after first human contact, and by implication very little is known about pristine life in marine waters, with the possible exception of the present-day ecosystems of the most southerly Antarctic waters, and the abyssal deeps. A better understanding of unexploited seas would serve as an important ecological baseline against which the impacts of human activities could be assessed.
- What is the relative importance of key drivers of environmental change over historical time frames? There is good evidence for several abiotic, climatic and human factors affecting life in the sea, but knowledge about their relative importance through time and their interactions remains poor.
- What has been the significance of marine resources for human societies over time? The physiological benefits of a marine diet are well documented but the economic and more intangible implications for historical societies have not been teased out.
- In cultural terms, societies have valued the goods and services provided by the sea very differently across various cultures and through time. What are the circumstances that have encouraged societies to exploit or give up the oceans?
- How may historical information be used for better ocean governance and management in the future? While there are examples of the usefulness of historical baselines for fisheries management, the wider potential of historical research for ecosystem and seabed management or the development of human coastal societies remains largely unexplored.

Beginning with an elaboration of these emerging research topics in the sections below, this paper aims to develop a new research agenda for marine historical research. We also discuss potential applications and further development of existing conceptual and methodological approaches, namely the ecosystem service concept, the use of indicators and modelling approaches, new molecular methods, advanced oral history, and the need for gendered historical research. We then discuss the current coverage of historical research, and suggest further expansion in terms of regions and ecosystems to ensure a true global perspective. Finally, we summarize our thoughts on the research agenda, and suggest the foundation of a global research network: the Oceans Past Initiative (OPI) to assist in coordinating research efforts worldwide.

## Impacts on Marine Systems Over Time

### 2.1 Untangling drivers of change

Little is known about what the oceans looked like before humans began to affect marine environments. The study of sediment cores as a library of past DNA provides one possible way of understanding what used to be swimming in the water columns above sea floors in a pre-exploitation era [3]. This could enable researchers to obtain a better understanding of pristine (pre-human) ocean life. It may also shed some light on when human activities started to impact marine systems, and how these effects can be differentiated from natural drivers of change. Similarly, a small handful of modern systems may yet be unaffected (or relatively so) by human impacts. These areas include the abyssal deeps, and the most southerly Antarctic waters [4]; the degree to which these provide clues about the functioning of other intact ecosystems prior to exploitation is currently unknown.

However, the vast majority of extant marine systems have been shaped by both natural and anthropogenic influences. Understanding the changes that these marine systems have undergone requires untangling the underlying drivers of these changes. Drivers are natural or human-induced factors that directly or indirectly force changes in ecosystems [5]. While direct drivers influence ecosystem components and processes themselves, indirect drivers alter one or more direct drivers. Physical and biological drivers such as changes in temperature regime or nutrient input, resource extraction, or the introduction of alien invasive species act directly upon marine systems and their component organisms [6]–[10]. Indirect drivers include human demographics, economic, sociocultural, and political processes, and scientific and technological innovation [11]. The majority of changes in ecosystems are caused by multiple interacting anthropogenic and/- or natural drivers. These interactions can be cumulative, antagonistic, synergistic, or otherwise. Decoupling the effects of single drivers to distinguish human influences from natural variability is a key interest of both scientists and ecosystem managers, and should be an integrative part of a research agenda that addresses historical change.

Determining impacts of natural drivers on pristine marine systems would improve our understanding of ecosystem functions and the role played by anthropogenic influences. Analysing the hard parts of organisms is one way to obtain information to infer the interaction of environmental drivers and ecological responses. For example, the shells of molluscs and otoliths (fish ear bones) are primarily constructed from calcium carbonate (CaCO_3_). Stable isotopes of oxygen, carbon and nitrogen have a long history of use as geological and biological tracers [12]–[14]. If fish otoliths, shellfish remains or other biological samples can be obtained from a known time in the past (e.g., from dated midden horizons, museum collections), then the stable isotope and band increment evidence for environmental temperature and/or organismal growth and maturation for that period can compared to the same measures from modern individuals to examine long-term change in parameters of interest, and potential effects of human activities. Stable isotope analysis can also help researchers to examine different aspects of climate, food web structures, and other characteristics of past marine ecosystems [15]. For example, sequences of temperature estimates based on δ18O in fish otoliths can be applied to infer season at capture, which contributes to a better understanding of past fishing patterns [16]. The technique has also been used for the study of marine species and human diet. Recently, Orton and colleagues have shown the growing role of cod imports for medieval London. Using cod remains from excavations, they proof the use of this technique for the indication of changes in human diet [17]. Naturally occurring climate variations have an impact on the productivity of marine ecosystems [18]. A number of investigations have documented substantial fluctuations in fish populations related to climate forcing [19], [20]. There is also well-established evidence for major climatic influences on phyto- and zooplankton communities [21], [22]. Recent studies have identified ecosystem-level shifts in various ecosystems around the globe [21], [23]–[25]. These shifts are a consequence of the interplay between climate forcing and various human impacts, primarily resource exploitation and pollution/eutrophication. Detailed investigations of reef growth and species composition in an Australian coral reef have shown that environmental degradation caused by human impacts has added additional pressure on the natural historical instability of these reefs [26]. Results from another Australian reef indicate that sediment and nutrient input following European settlement prohibited the recovery of *Acropora* assemblages after a series of acute disturbance events (SST anomalies, cyclones and flood events) [27]. One approach to detangling the impacts of such combined natural and anthropogenic drivers is the examination of paleontological, climatological, archaeological and historical evidence from localities affected by similar climate events but with differing levels of human impacts [28]. Such an approach could also provide a fresh perspective on natural variability.

#### Required research

Untangling natural and anthropogenic causes of environmental change to estimate the extent of human impacts on the oceans should be one of the major tasks for historical research in the near future. Coordinated multi-disciplinary research activities around the globe that focus on the same time period in areas of contrasting human exploitation of marine resources will assist in distinguishing the effects of natural and human drivers of marine environmental change.

### 2.2. The role of humans

Examination of the earliest human interactions with the marine environment in most parts of the world is beset by a major problem. The rise in sea level after the most recent ice-age has obscured or destroyed much of the earliest evidence of human exploitation along the margins of ancient shores [29]. Although in some areas such as the North Sea some evidence of early exploitation has been recovered from submarine deposits it has often been reworked by waves and currents making interpretation difficult [30]. However, there is evidence for an intensive use of marine resources by early modern humans in Pinnacle Point (South Africa) some 164,000 years ago. Gathering shellfish seems to have been part of a response to sea level fluctuations, which forced these people to expand their home ranges and to follow the shifting position of the coast [31]. Almost contemporarily, Neanderthals gathered shellfish in the coastal waters of Torremolinos in Southern Spain [32]. Archeological studies have revealed a general shift in marine resource exploitation from inshore towards offshore areas. Marine fish became increasingly important - as early as 1,500 years ago - on the Californian coast [33]. Similar patterns have been discovered in the Wadden Sea [34].

But for most of human history, the extent of anthropogenic impacts on natural environments was largely restricted to local and regional levels. Within the last 200 years, human actions reached the global level by affecting processes such as global nutrient and water cycles. The traces of human activities are not restricted to populated areas anymore, but can be found everywhere in the ocean [35]. For example, plastic debris is accumulating at the shorelines of even the most remote islands and in the deep sea [36]. Johan Rockström and colleagues [37] claim that human actions have accelerated to such an extent that they might push the Earth beyond its “planetary boundaries”. The term planetary boundary refers to thresholds between alternate states of the global system. Once crossed, the system enters a new state, and irreversible environmental change might be unavoidable. By now, the existence of thresholds has been posited for numerous ecological and social-ecological systems, including the marine realm (see the thresholds database of the Resilience Alliance and the Santa Fee Institute, http://www.resalliance.org/index.php/thresholds_database). The world has entered a geological new era: the Anthropocene.

In this era, an important line of research will be the study of the interaction of different human activities and their impacts on specific marine areas in the past. Cross-regional comparisons of magnitude and direction of these changes to marine life in response to multiple human pressures could also be extremely helpful in teasing apart multiple long-term drivers. For example, it was only by the late 1990s that more or less the full range of harvestable fish and invertebrates in all ecological strata of Southeast Asian waters was caught [38]. Near-pristine marine systems could still be found in some parts of the Indonesian Archipelago until the 1960s, a time at which Atlantic waters had already been affected by industrial fishing activities for more than two centuries. In particular, data from large, isolated, late settled islands or regions such as Madagascar, New Zealand, and Antarctica, where significant human impacts became relevant much later than in the seas bordering the major continental landmasses, can play an important role in distinguishing among natural climatic variability and human drivers (including anthropogenic climate change). Recent work in Australian coral reefs has shown that it is indeed possible to differentiate natural variability from human-induced environmental change [27].

Despite the importance of similar drivers acting at the global level, most ecosystem changes are caused by a set of interactions that are more or less unique to a particular place [11]. To understand how resource and socio-economic endowments, in combination with other factors, have created specific exploitation patterns, bioregional histories of marine environments need to be developed. This will also provide a more comprehensive view of “what once was”, e.g., historical baselines of marine animal populations, enhancing our ability to articulate desirable ecological states and management recommendations [39].

In this line of research, specific attention must be paid to the role of globalisation and changing consumption patterns. Processes of globalisation have had a significant influence on marine resource exploitation for hundreds of years. The quest for marine resources was an important aspect of European expansion into Asia, Africa and the Americas. Arctic marine mammals, sea turtles in the Caribbean, and cod from Iceland to Newfoundland are just some examples of valuable sought-after species [40]. Similarly, Asian, particularly Chinese food and medicine markets, have driven a search for marine species on a regional to global scale over the past few centuries. For example, the development of sea cucumber fishing and trading by people from Makassar, Indonesia, can be traced from its beginning more than 300 years ago to the industrialization of the fishery in the 20^th^ century and the depletion of sea cucumbers in the 1980s [41]. Economic gain was only one side of the story. Changing preferences for certain products, the exploitation of new areas, and technical innovations all played a role in a widening search for marine resources.

#### Required research

How has the pattern of marine resource exploitation proceeded in each area settled by humans, and which species were affected? Was the unfolding of events in Africa fundamentally different from histories in Asia, Australia, the Americas, and Europe and recently settled remote islands? To address this will require integration of the earliest archaeological records with historical and modern information sources to determine patterns of marine resource use over the entire period of human settlement of coastal margins. Studying the role of globalisation and changing consumption patterns may improve our understanding of resource use by linking serial depletion on a global scale to local demands. We also need research into changing consumeŕs preferences, especially towards more sustainably harvested marine products. Do such changes occur on a short or long-term scale, are they caused by public advice, by economic forces, or by other cultural factors that are to date poorly understood?

## Altered Marine Ecosystems

### 3.1. Extinctions, species declines, and habitat changes

Extinctions and resource depletions in the marine realm have been evaluated by a number of studies (eg., [41], [42], [43]). We now have a relatively precise overview of globally extinct marine mammals, birds, fish, and molluscs [42]. Many species have become extirpated, or nearly so, across part of their range, such as is the case with several shark and ray species in the Mediterranean Sea [43]. Others have been depleted to such an extent that they cannot fulfil their former role in the ecosystem, as in the case of reduced abundances of some filter feeders [44]. To truly understand the extent of these changes, long-term biodiversity loss should be studied in different regions of the oceans over various periods of time. This includes the reconstruction of decadal, centennial, and millennial dynamics of marine animal populations [45], [46]. A number of studies have provided important knowledge in this respect, for example on changes to shark populations in the West Atlantic [47], or on numbers of large predatory fish in different seas [48]. Most of the historical studies to date have concentrated on exploited higher trophic levels of marine animals, while intermediate and lower trophic levels have largely been ignored [49]. In large part this is because the evidence about large exploited species is more readily available from archaeological and historical sources. But there is also evidence for a sequential exploitation of invertebrate species in the past [41]. How this has shaped the status of ecosystems should be an important research topic in future.

Changes in coastal and marine habitats are a related research topic that deserves more attention. Although this line of work has been less prominent that changes in species, important contributions have already been made. For example, Lotze and colleagues reconstructed time lines, causes, and consequences of change in several estuaries and coastal seas worldwide and found similar patterns of habitat destruction, water quality degradation, and the influence of invasive species [44]. Other work has also focused on sub-tidal algae forests in the Adriatic Sea [50], or provided a two-century perspective on changes in a Scottish coastal area [51].

#### Required research

In the future, a major research avenue may be to link geospatial data and historical maps showing coastal and marine habitats to information about past distribution and abundance of species derived from archaeological, historical, and genetic sources. Such research could help us to understand indirect causes and effects of habitat change and be of value to marine and coastal management. Focussed research on species across levels, including intermediate and lower levels, is required to describe the patterns and magnitudes of their exploitation, preferably from the period of first human settlements to the present day.

### 3.2. Recoveries of populations, habitats and ecosystems

There are many examples around the world where previously affected species, habitats and ecosystems have or are responding to new management interventions. For example, over the last 40 years of protection populations of New Zealand fur seal (*Arctocephalus fosteri*) have started to rebound after 600 years of exploitation first by Polynesian settlers and later by European sealers [52]. Interestingly, the same protection regime has made little impact on the recovery of New Zealand sea lions (*Phocarctos hookeri*). In this species the return of maturing females to their beach of birth has limited the expansion of breeding populations into their historic range [53].

Research on ecological recoveries has a very clear link to providing input to decision-makers and managers, because it brings a historical perspective into the present management of marine resources and tells them what has worked so far and what not. Given that the approach to managing marine systems is evolving towards ecosystem-based adaptive management, a long-term, historical perspective to ecosystem function and change is very much needed [54], [55]. An interesting example of such work is a recent reconstruction of social-ecological interactions over the last 700 years in Hawaiian coral reefs. It reports previously undetected recovery periods related to a complex set of changes in underlying social systems resulting in the release of reefs from direct anthropogenic stressors [56].

#### Required research

Future investigations should continue to assess the adaptive capacity and resilience of degraded ecosystems and depleted stocks to recover from human impacts. There is already work done on how marine resources have recovered from previous exploitation-related declines [45], [57], but we need a stronger focus on which species, ecosystem traits, and management activities might facilitate recoveries.

## Perceptions, Governance, and Management of Marine Systems

How people perceive and value marine environments and the resources they provide determines individual and collective preferences, actions and strategies in the marine realm. Historical descriptions of coastal and marine environments are prime examples of documented perceptions of the past. In the absence of other data, they have been successfully used to establish abundance changes of marine species in different time periods [58], [59].

Different perceptions and valuation systems also underlie the institutional structures that govern and manage marine systems. Research on governance structures must be linked with research on perceptions and values to understand what drives and has driven approaches to marine resource exploitation and its management in different periods of time. For analytical clarity, it is important to distinguish between governance and management. Governance describes a social function centered on efforts to steer the actions of humans toward achieving desirable outcomes and avoiding undesirable ones. It covers the fundamental goals, institutional processes and structures which are the basis for planning and decision-making, and sets the stage within which management occurs. Management refers to the process by which human and material resources are used to achieve a defined goal within a known institutional structure [60].

The notion of the sea as a seemingly endless source of resources has long dominated marine governance, or rather the relative absence of institutions governing the sea. However, there are also examples of long-enduring traditional management regimes which have regulated the exploitation of valuable or scarce resources such as pearl oysters, sea cucumbers, or *Trochus* spp. shells in many coastal areas in the Pacific and parts of Southeast Asia, long before modern fisheries management was invented. For example, the Indonesian regime of “sasi laut” regulated access to marine resources by placing temporal and spatial harvesting restrictions on them. Similarly regulations in European inshore waters such as the Limford sustained fisheries for hundreds of years [61]. While such community-based management is certainly not a panacea, studies around the world have found that traditional community-based governance regimes contain elements that may support sustainable resource use [62], [63].

### 

#### Required research

How have different local perceptions of marine systems affected their governance and management, and how have these evolved over time? Can we identify elements of these historical, community-based approaches to ocean governance and management that are also relevant to modern marine management?

## Emerging methodological approaches

A number of emerging methodological approaches are expected to have a considerable influence on future marine historical research. They include conceptual developments such as the application of the ecosystem service concept in historical analyses or the consideration of social issues like gender and equity issues, but also new biophysical tools, for example different kinds of molecular and stable isotope analyses. These approaches are not necessarily new to science, but it is their application to historical research that is expected to improve our ability to analyse and evaluate changes in marine systems and their organisms over time.

### 5.1 Applying the ecosystem service concept in historical analyses

Ecosystem services are an array of potential benefits derived from specific ecological components and processes, ranging from the provision of fish to the capacity of the ocean to buffer climate change. Environmental change has clearly altered the level of ecosystem services provided by marine systems, but to what extent is largely unknown. There is a need to improve our understanding of how past changes in marine systems have affected marine ecosystem productivity and marine ecosystem functioning, and how this has influenced the ability of marine systems to provide ecosystem services.

The ecosystem service concept values nature in relation to human uses. By acknowledging the role of ecosystems as providers of essential goods and services, it links ecosystem functions with the economy and social spheres, including livelihoods and well-being [64]. The ecosystem service concept thus provides an approach to assess the value of marine systems, although this does not have to be a monetary valuation. More importantly, it offers a standardized method to compare these values over time, by showing how changes in ecological functions have caused changes in benefits to society [65].

Although much historical research is clearly related to ecosystem services and benefits, the concept of ecosystem services itself has hardly been used in the discipline. One exception is the decline in marine mammals in New Zealand that followed the well-documented onset of Māori sealing soon after initial settlement, and European whaling in the early 19th century [66]. Both cultures viewed many species of marine mammals as valuable commodities to be harvested, so the numbers of these mammals declined precipitously as a result [67], [68]. Now, these species are protected and their value to New Zealand as a provisioning service has declined to zero. Instead, marine mammals are currently prized for their spiritual and existence value; people enjoy directly viewing them from land, sea, and air; and they are appreciated as subjects for research and educational activities. Moreover, whales are now recognised as having important roles in ecosystem regulation [69]. Studies like these illustrate the usefulness of the ecosystem service concept as a common framework for analysing the impacts of environmental change on societies.

### 5.2 Developing indicators

Indicators are characteristics of ecological and social systems or their components, such as species, populations, networks, and social groups, which ideally indicate a certain state or dynamic of a system that is otherwise difficult to measure and evaluate. Because ecological and social systems are inherently complex, the use of indicators helps to describe them and their changes in simpler terms. If chosen well, indicators can track changes over time and across species or regions, and can inform managers and the general public.

One of the most fundamental ecological indicators of historical change is a change in population abundance. This can be measured as a decrease or increase in the number of individuals, their biomass, average size or age, as well as an expansion or contraction of their distribution over time [70]. These indicators have been applied to a variety of records from the past. For example, archaeological records from shell middens revealed declines in the relative abundance, size and age of white sturgeon from 2600 to 700 years ago based on their bone frequency and dentary width [71], [72]. Historical whaling maps and log books have been used to reconstruct the rapid depletion of right whales (*Eubalaena japonica*) in the North Pacific by 19th-century whalers [73]. Fisheries catch and effort data throughout the Mediterranean have been used to analyse declines in the catch-per-unit-effort (CPUE) of sharks since the 19th century [74]. In rarer cases, it has been possible to extend such analysis into the early 17^th^ century [75]. Other fundamental ecological indicators are the basic presence or absence of a species in an ecosystem, the trends in functional groups, or changes in trophic habits and positions [70]. Future development of ecological indicators will move towards standardization and evaluation of indicators, and the development of reference levels (targets, thresholds, limits) to inform the management of marine resources and ecosystems.

Social indicators are expected to play a very important role in the future for the assessment and management of coastal and marine systems and their changes over time. Such indicators can be qualitative or quantitative and are used to describe the status of social systems, and their dynamics and processes. While status indicators measure, for example, the current perception of target species, their availability or the social networks of resource exploitation, process indicators assess specific actions, changes or functions over a defined time period, such as participation, conflict resolution or institutional change [76]. Social process indicators can be used to evaluate participation and decision-making in the management of coastal and marine resources by assessing metrics such as the number of meetings, the levels of participation, and the character of social networks involved.

Indicators are expected to play a far greater role in the analysis of social–ecological systems in the future. They allow for regular measurements of key ecological, socio-economic, and social–ecological processes to better understand system changes and their underlying causes [76].

### 5.3 Utilizing modelling approaches

A major research issue in this field is how to integrate historical knowledge and ecosystem modelling, including past and future applications and simulations. The future will bring developments in modelling techniques which increase the capability to hindcast ecosystem dynamics and ensure forecasting possibilities [70]. More comparative analysis of qualitative and quantitative models must be developed since the ability to model past marine ecosystems is crucial to understanding the present, and to predict the future. These analyses will be challenged by the urgent need to consider ecological, economic and social dimensions of change [70].

Qualitative and quantitative modelling techniques [77]–[81] have already been adapted or modified to model past marine food webs. For example, qualitative modelling was used in the Gulf of Maine to identify distinct and sequential phases in the trophic structure of kelp forests [82]. The authors used archaeological, historical, ecological, and fisheries data to document a serial targeting and depletion of abundant top consumers that led to functional loss of trophic levels, creating trophic cascades that changed the structure and function of the ecosystem [83]. In the Adriatic Sea, qualitative modelling has similarly been used to describe the historical change of marine food webs using ten historical periods that started in the pre-human period before ∼100 000 BC to the global expansion of humans in AD 1950–2000 [84].

An interesting approach to modelling past ecosystems has been developing under the “Back to the Future” approach [85]. This approach aims at evaluating historic ecosystems as tools to define possible restoration goals and to design rebuilding strategies for ecosystems. Two remarkable applications were developed for Canadian marine ecosystems of Newfoundland [86] covering the years of 1450, 1900, 1985 and 1995, and in British Columbia [87], covering the periods of 1750, 1900, 1950 and 2000. Both studies highlighted the general depletion of marine resources since the first European contact and the important changes in the structure and functioning of food webs through time.

### 5.4 Application of molecular tools

Molecular genetics has fundamentally changed our understanding of marine ecology. Investigations have demonstrated extensive adaptive change in marine populations in response to natural and anthropogenic drivers. Research has also shown that the estimated population sizes of several species are much smaller than census sizes, which is highly relevant for management and conservation [88]. A notable example of the use of molecular techniques in historical ecology studies is Roman and Palumbi’s study on North Atlantic humpback whales (*Megaptera novaeangliae*), fin whales (*Balaenoptera physalus*) and minke whales (*Balaenoptera acutorostrata*), in which they used molecular markers to estimate the pre-exploitation abundance of these species. Their results indicated that current population numbers are far below their original size [89], (although, in a recent overview, Palumbi was careful to outline the indefinite time parameter of their study and a number of uncertainties [90]). A genetic approach was also used to estimate pre-whaling abundance of Eastern Pacific gray whales. The results substantially differed from population reconstructions based on catch records, which had considered the population to be fully recovered [91]. Both examples clearly demonstrate the relevance of molecular tools in defining baselines for management of marine species, and the recovery of depleted populations.

Molecular tools also have an enormous potential to contribute to the discussion on detangling natural and anthropogenic drivers of change. A recent study using genetic data from the harbor porpoise (*Phocoena phocoena*) in the Black Sea was able to reconstruct the demographic expansion of this species some 5,000 years ago as a result of natural environmental changes, as well as its population collapse due to recent anthropogenic activity [92].

Molecular analyses are increasingly used in research on biological invasions, one of the most serious human-induced threats to marine ecosystems [49]. This has improved our ability to make inferences regarding invasion histories, enabled resolution of some of the long-standing questions regarding the cryptogenic status of marine species and provided means to recover the patterns of community structure of the ocean’s biota.

### 5.5 Advancing methods for written documents and oral history

The provenance of documents traditionally frames modes of inquiry in the history discipline. Port records, log books, tax ledgers etc. are typical sources of quantitative information, while interviews, written memories or newspaper coverage can provide both quantitative and qualitative information.

Witness testimonies, especially, are increasingly used to acquire information on past and contemporary marine environments and fisheries. Through testimonies, individuals or groups share their perceptions and opinions about past events or experiences. Oral history helps to gather these accounts of the past, and to make use of traditional and local environmental knowledge that resource users have accumulated [93]. If oral histories have been recorded or written down, the time span for which this kind of data can be used extends far beyond living memory. The scientific literature on the use of oral history has grown rapidly over the last three decades [94]. But after Saenz-Arroyo’s pioneering study on shifting baselines in fishermen’s memories [95], the work by Palomares and colleagues on the establishment of abundance data from historical narratives [58], and the application of a fuzzy logic approach to data gathered from interviews [96], the methodological progress seems to have slowed, although the value of oral history has been clearly demonstrated [59].

An important aspect which certainly deserves further attention is the creation of quantitative data. Qualitative information is typically rich and insightful but difficult to control for selectivity and representativity, a problem at the very heart of the historical discipline which emphasises the critical work of uncovering problems of bias. A basic social science approach – the coding of qualitative data – can provide an important contribution in this respect. Coding comprises of the search for emerging topics and key words in documents or interview transcriptions, and their description with a unique code. This allows, for example, quantitative frequency analysis of topics.

In recent years, discourse analysis, meta-analysis and digital representation have provided historians with a wide range of new methodological and technical tools to grapple with vastly increased masses of data. “Big Data” tools which organise masses of data for geographical, temporal and discursive analysis has the potential to bring historical interpretation to a new level of breadth and precision. Marine environmental history has not yet fully embraced the potential of such new methodologies. In particular there is great potential in the application of database and geographical information tools for the study of disparate oral and documentary data.

### 5.6 Using a gender lens

Marine resource exploitation in general, and fisheries in particular, are often perceived to be a male domain [97]. This is largely the result of the socially constructed roles of men and women within societies: while men are typically regarded as providers, women take care of the home and family. It has also to do with the traditional perspective that fishing refers to the catching of fish with specific gears, such as lines and nets. Gleaning from shorelines and reefs has rarely been acknowledged as fishing [98]. Additionally, it has often been assumed that fisheries largely operate in the public domain, a usually male dominated sphere. The rather female dominated private domain is mostly not in the focus of attention. But especially in subsistence and other small-scale fisheries, much of the administration and logistics including financial issues, as well as the processing happens in the household and through family networks. An analytical focus on public and formal practices misses women’s roles as well as a considerable part of what it takes to organize and put into practice a fishing operation [99].

This has two major implications: ignoring the role of woman in fisheries can lead to a substantial underestimation of fishing pressure, especially in coastal areas. It also leads to an underestimation of the social and economic contributions that women provide in fisheries, especially in processing and other value-adding activities [100]. A gender lens can increase understanding of the history of marine resource exploitation. Differentiating roles, responsibilities, access and opportunities of men and woman will provide a more complete picture about access to and control of marine resources [101].

Since the first consolidated publication on women in fisheries by Nadal-Klein and Davis [102], the literature has increased rapidly. While some of these publications have focussed on the role of women in marine resource collection [103], [104], pre- and post-harvesting activities [105], or their role in governing and managing marine resources [106], [107], others are rather conceptual contributions such as Yodanis’s work on the social construction of gender in fishing communities [108]. Recent work has also high lightened women’s in depth knowledge on species and environmental changes. For example, oral histories in New Zealand indicate the important role that women, often accompanied by children, once played in gathering seafood from shallow, easily accessible shores sixty to seventy years ago [109]. Women’s knowledge confirmed that fish and shellfish were very abundant and could be reliably caught or gathered with little effort.

Another line of work has looked at masculinity and its linkages to fishing. For example, Fabinyi [110] has described how illegal fishing activities in the Philippines provide young men with a higher social status, while Allison has argued that physical settings and the distinct culture of fishing societies shape similar “marine masculinities” in fishing communities in different regions of the world [111]. In a recent publication, Schwerdtner Máñez & Pawelussen [99] call for a gendered perspective on the different roles of both men and women in order to better understand the history of marine resource exploitation over time.

## Coverage of Historical Analyses

Under the auspices of HMAP, twelve regional and three species-based projects were developed between 2000 and 2012. These uncovered a wealth of historical information that has been published in more than 200 peer-reviewed scientific publications. However, even such a concerted effort could by no means attain a global coverage in the investigation of humankind’s interactions with the sea. To understand the history of human interactions with life in the oceans on global and smaller scales historical research needs to be extended to new geographical areas.

Costello and colleagues have identified a number of areas that received little or no attention during the Census of Marine Life, such as the southern and eastern Mediterranean Sea; estuaries, coastal areas and coral reefs of the Indian Ocean; large regions of South America and the Indian Ocean; and many other habitats including ice-bound waters, ocean trenches, and some deep sea areas [49]. In terms of historical investigations, this list of gaps is even longer.

South America is one of the regions which have so far received very little attention. Although there are an increasing number of studies on the environmental history of the continent, these have had an almost exclusively terrestrial focus. One of the noteworthy exceptions is the work by Perri, who examined the Spanish overexploitation of the oyster beds around the island of Cubagua in what is today Venezulea [112]. Other work covers the ecological history of the Peruvian guano industry from 1800–1973, and its relation to the development of world’s largest industrial fishery, that of anchoveta [113]. More recently, Eddy et al. have studied the change in lobster biomass over 400 years of human fishing activity on the Chilean Juan Fernández archipelago [114]. But the vast majority of the continent’s coasts and marine areas remain untouched by historical studies.

For the African continent, the situation is similar. An exception is South Africa, where a number historical studies have been undertaken, including work on long-term trends on commercially exploited species [115], [116], and work on the history of harbors and fisheries [117]. In an overview of the history of marine and estuarine studies in South Africa, the author argued that South Africa has some of the best data in the world on the biodiversity of rocky shores, coastal waters and estuaries exists, as well as large collections of many taxa [118]. Clearly, further historical work could take advantage of these rich collections. There are also some historical studies on the island of Rodriguez, including research on shifting baselines [119], [120]. But for the rest of the continent, we are not aware of any studies in marine environmental history or historical ecology.

The polar regions certainly represent another region with a rich history in marine resource exploitation: whaling, seal hunting, and search for fish have brought people for centuries into these areas. The history of whaling has received some attention, for example in a paper by Hacquebord who analyzed the impacts of eliminating thousands of whales and walruses on other marine mammals [121]. Other studies have assessed ecological changes or population trends resulting from the mass removals of pinnipeds and whales [122]. Whaling records have also been used to establish declines in Antarctic sea-ice extent [123], and the influence of environmental factors on whaling operations has also been studied [124]. However, much research could still be done in these regions to expand our understanding of how they have been changed by resource extraction over time.

An area for which we have very little information is the third dimension of the oceans: the deep sea [125]. Whereas the coastal areas of most developed countries have been exploited for several centuries, the deep sea waters were little known by fishermen and marine scientists alike just 100 years ago. In the meantime deep sea investigations such as the Dutch Snelllius Expedition of the 1920s, and multiple Danish and Norwegian led deep sea investigations traversed the Atlantic, Indian and Pacific Oceans from the turn of the 20^th^ century until the 1960s. *The Carlsberg Foundation’s Oceanographic Expedition Round the World* from 1928–30 is an example of how global biogeography was charted well in advance of real impacts from resource exploitation in the deep seas of the World’s oceans [126]. Today many of these areas are exploited for multiple marine resources. Such early endeavours deserve careful scrutiny to provide reference points for present day ocean management.

Another focus of attention should be on historically data-rich areas. For example, much documented evidence is available for large parts of maritime Southeast Asia, including the coral triangle. This region is the centre of global marine biodiversity, and the exploitation and trade of its marine resources has at least partly been documented by its former colonial regimes. Especially for the former Dutch East Indies, but also for what today is Papua New Guinea, a wealth of historical material has been collected in Dutch and German libraries and archives, and its processing has just begun [127]. Japan and China are also countries with a long tradition of record keeping, and strong connections to the sea. Working with these data might also improve our understanding of early drivers of resource exploitation, which created networks and trade routes that are still important today [41].

## Outlook

Archaeological and historical research has still much to contribute to an improved understanding of the development of marine and coastal ecosystems, and their interactions with humans over time. It helps not only to identify patterns and trends, but also to quantify amounts of living marine resource exploitation. While the aim of this paper has been to establish a new research agenda, we would also like to point out that new institutional arrangements are required in order to successfully implement it. To complement individual projects, which end when their funding runs out, we suggest the establishment of a global research network for marine historical research: the Oceans Past Initiative (OPI). This could become a venue where researchers worldwide interested in historical studies could meet and discuss relevant issues virtually. OPI could provide an umbrella, under which already completed, currently running, and also planned projects and initiatives could be linked, and make their results available for decision-makers and the interested public. Such a network could also commit itself to coordinate resources and provide useful information to the research community, such as by circulating news about grant calls and funding opportunities, information on recent papers in the field, announce successful projects, and distribute information for students on courses related to marine historical research. A first major step into this direction has been undertaken through the establishment of the Oceans Past Platform (OPP), a COST action supported by the European Union which aims to measure and understand the significance and value to societies of living marine resource extraction and production to help shape the future of coasts and oceans.
